# Potassium Thiocyanate‐Assisted Enhancement of Slot‐Die‐Coated Perovskite Films for High‐Performance Solar Cells

**DOI:** 10.1002/smsc.202000044

**Published:** 2021-02-07

**Authors:** Fuzong Xu, Jiang Liu, Anand S. Subbiah, Wenzhu Liu, Jingxuan Kang, George T. Harrison, Xinbo Yang, Furkan H. Isikgor, Erkan Aydin, Michele De Bastiani, Stefaan De Wolf

**Affiliations:** ^1^ Physical Sciences and Engineering Division (PSE) KAUST Solar Center (KSC) King Abdullah University of Science and Technology (KAUST) Thuwal 23955-6900 Kingdom of Saudi Arabia

**Keywords:** hysteresis of perovskite solar cells, perovskite grains, potassium thiocyanate additive, slot-die coating

## Abstract

Slot‐die coating is highly promising for scaled deposition of metal halide perovskite thin films. However, the power conversion efficiencies (PCEs) of slot‐die‐prepared perovskite solar cells (PSCs) still lag behind their spin‐casted counterparts. To resolve this issue, the crystal size and quality of slot‐die‐coated methylammonium lead triiodide (MAPbI_3_) perovskite films are dramatically improved via additive engineering using potassium thiocyanate (KSCN). The modified micrometer‐thick films have an average grain size of ≈11 μm and charge‐carrier parameters that are comparable with single‐crystal perovskites, such as a 1.89 μs lifetime, 136.65 ± 31.52 cm^2^ V^−1^ s^−1^ mobility, and 25.15 ± 3.55 μm diffusion length. Exploiting these enhanced properties, planar inverted PSCs with negligible hysteresis are fabricated and an average and a maximum PCE of 20.14% and 21.38%, respectively, are achieved which are among the highest reported values for slot‐die‐coated PSCs. Notably, our devices have a narrow PCE distribution along the slot‐die coating axis, highlighting slot‐die coating's promise to fabricate large‐scale, high‐performance PSCs.

## Graphical abstract

Assisted by potassium thiocyanate (KSCN) as additive, slot‐die‐coated MAPbI_3_ perovskite films are prepared with ultra‐large grain size of up to 11 μm and remarkable charge‐carrier diffusion lengths up to 25.15 μm. Using these films, perovskite solar cells with a power conversion efficiency of 21.38% with negligible hysteresis are obtained with a device stack entirely fabricated using scalable methods.

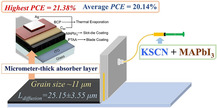

## 1 Introduction

In recent years, the record power conversion efficiency (PCE) of lab‐scale perovskite solar cells (PSCs) has rapidly increased to 25.5%.^[^ ^1^ ^]^ However, currently, the perovskite films used in such high‐performance devices are usually deposited by spin coating, which might not be suitable for square‐meter scale manufacturing.^[^ ^2^, ^3^, ^4^, ^5^ ^]^ To fulfill the promise of perovskite photovoltaic technology, it is imperative to combine high performance with affordable cost, underlining the urgency of translating these results to high‐throughput scalable fabrication techniques. Several scalable solution‐deposition methods can be considered toward this goal, such as spray coating, electrochemical deposition, soft‐cover method, blade coating, and slot‐die coating.^[^ ^6^, ^7^, ^8^, ^9^, ^10^, ^11^ ^]^ Blade coating has already been successfully used for PSCs;^[^ ^12^, ^13^, ^14^, ^15^, ^16^ ^]^ however, the technique is not ideal to realize truly scalable large‐area deposition due to the lack of continuous ink supply. Slot‐die coating resolves this issue and is a proven technique for roll‐to‐roll printed organic solar cells.^[^ ^17^, ^18^, ^19^ ^]^ However, to date, slot‐die‐coated PSCs’ performance still lags behind that of spin‐coated devices. The rapid deposition of perovskites using slot‐die coating often causes small flower‐like grains with significant pinholes and a considerable amount of grain boundaries and defects.^[^ ^10^, ^11^, ^20^, ^21^, ^22^ ^]^ These imperfections are detrimental to the optoelectronic properties and stability of perovskite films, and accordingly, impair the performance of PSCs.^[^ ^23^, ^24^, ^25^ ^]^ Therefore, to enable high‐performance devices using high‐throughput methods, deposition of perovskite layers with large grains (and minimal grain boundaries in the transverse direction), as well as fewer defects, is of critical importance.

Reportedly, recrystallization and Lewis base–acid interactions can enlarge perovskite grains and eliminate defects; moreover, earlier reports indicate that potassium can effectively passivate surface defects.^[^ ^26^, ^27^, ^28^, ^29^, ^30^, ^31^ ^]^ To obtain these stated effects simultaneously, we use here potassium thiocyanate (KSCN) as an additive in methylammonium lead triiodide (MAPbI_3_) precursor solutions. Moreover, to lower the processing temperature, aimed at protecting the underlying poly(triaryl amine) (PTAA) hole transport layer, we use in this work the methylamine (MA)/acetonitrile (ACN) complex solvent with low boiling point, rather than the conventional dimetyl formamide (DMF)/dimethyl sulfoxide (DMSO) mixture, which has a higher boiling point.^[^ ^32^, ^33^ ^]^ We find that the KSCN additive enlarges the grains significantly (with average diameters up to ≈11 μm) and reduces defect densities, thereby enabling slot‐die‐coated MAPbI_3_ films with long carrier diffusion lengths (25.15 ± 3.55 μm). The effect of this additive appears to be much more pronounced here, using slot‐die coating, compared with earlier reported results with spin coating.^[^ ^34^ ^]^ We also observe a slightly red‐shifted (i.e., narrower) bandgap upon KSCN utilization. The long charge‐carrier diffusion length, comparable with that of high‐quality single‐crystal perovskites in previous studies,^[^ ^35^, ^36^ ^]^ allows for efficient charge–carrier collection even in micrometer‐thick PSCs, as used in this study. The use of such thick films also results in improved absorption of the available photons.^[^ ^37^, ^38^, ^39^ ^]^

Consequently, due to enhanced optoelectronic properties, the short‐circuit current (*J*_sc_), open‐circuit voltage (*V*_oc_), fill factor (FF), and the PCE of our devices are significantly improved. We also observed a reduced hysteresis factor (HF) and enhanced device stability with KSCN addition. Upon testing our PSCs with an aperture mask, we demonstrate a maximum PCE of 21.27% (average of 20.14%). We show high‐performance slot‐die‐coated PSC with comparable PCE values as state‐of‐the‐art lab‐scale devices with these advancements. Notably, we observe almost identical PCE values for the multiple pixels distributed over a large area, underlining further the promise for scaled manufacturing of high‐quality perovskite thin films.

## 2 Results and Discussion

**Figure 1:**
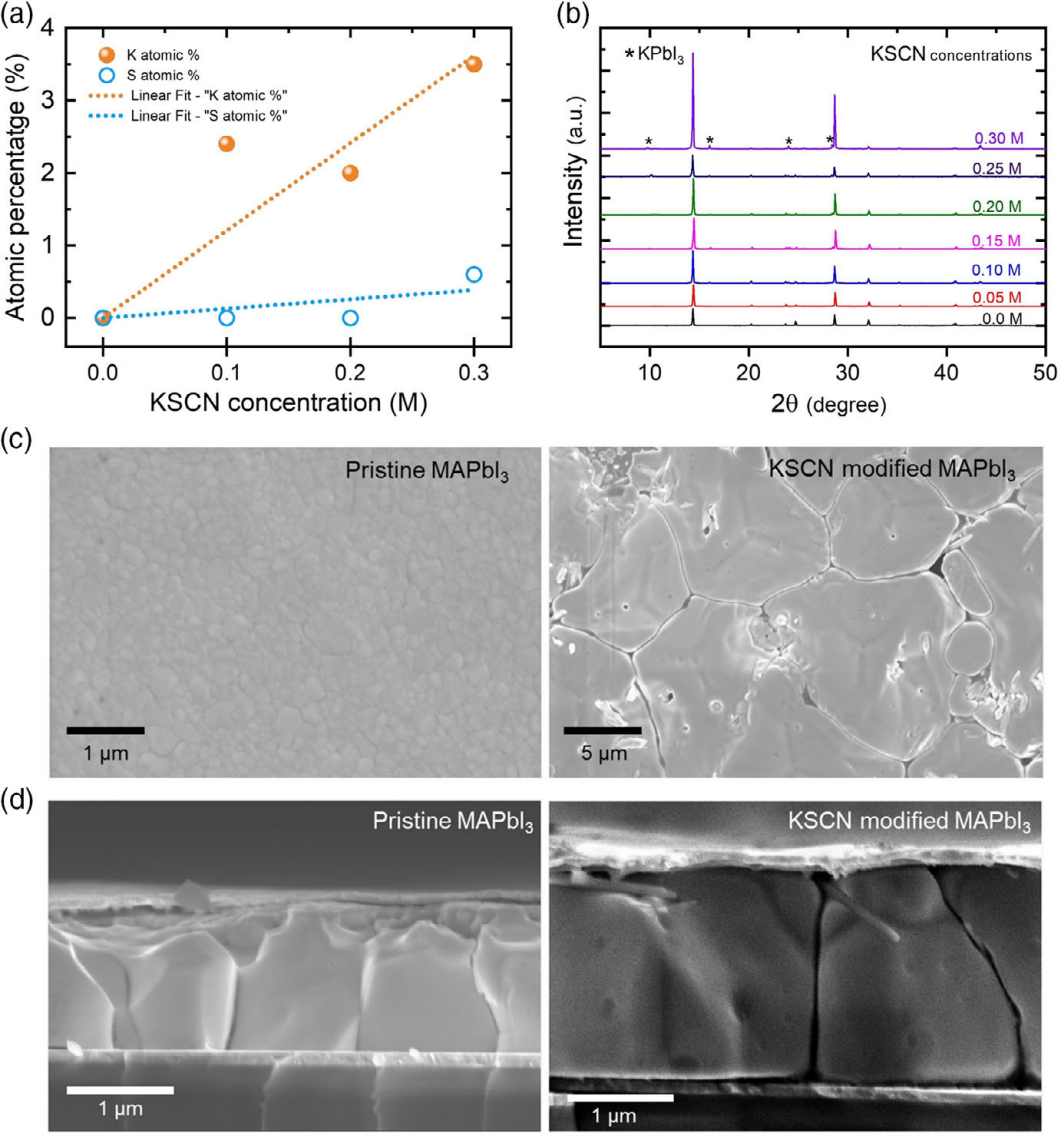
Structural, elemental, and microscopic characterizations. a) The atomic percentage of K and S in films supported by XPS in Figure S1. b) XRD patterns for perovskite films deposited by MAPbI_3_ precursor solutions doped by KSCN with a concentration from 0 to 0.3 m with 0.05 m steps. c) Top‐view and d) cross‐sectional SEM of pristine and KSCN‐modified MAPbI_3_ films deposited on PTAA.

In our work, we compared a simple MAPbI_3_ absorber fabricated using slot‐die coating, with and without KSCN additive in the planar p–i–n device architecture. Chemical reactions triggered by the KSCN additive during perovskite film formation are evident from X‐ray photoelectron spectroscopy (XPS) (**Figure** **1**a) and X‐ray diffraction (XRD) (Figure 1b) results. As shown in Figure 1a and Figure S1, Supporting Information, the XPS results (K2p and S2p core levels) show that the potassium (K) concentration is directly proportional to the concentration of added KSCN. Still, the concentration of sulfur (S)—indicative for the presence of SCN^−^—is negligible. The measurements indicate that after perovskite film crystallization, K^+^ cations remain in the perovskite films, but SCN^−^ anions do not. The XRD results (Figure 1b) confirm that K^+^, as the concentration of KSCN increases, is incorporated in the form of KPbI_3_ crystallites. Especially over concentrations of 0.10 m, the diffraction peaks of KPbI_3_ appear with increasing intensity.^[^ ^40^ ^]^ These results are consistent with scanning electron microscopy (SEM; Figure S2, Supporting Information) and atomic force microscopy (AFM; Figure S3, Supporting Information) images: the number of rod‐like crystallites, associated with the appearance of the orthorhombic KPbI_3_ crystal structure, increases with KSCN concentration.^[^ ^40^ ^]^ Based on these results, the reaction below shows the proposed mechanism of KSCN interaction in the formation of MAPbI_3_.

(1) $${\text{MAPbI}}_{3}(\text{solution})+x\text{KSCN}(\text{solution})⇌x\text{MA}(\text{gas})↑+x\text{HSCN}(\text{gas})↑+x{\text{KPbI}}_{3}(\text{solid})↓+(1-x){\text{MAPbI}}_{3}(\text{solid})↓$$

Moreover, as the concentration of KSCN increases, the XRD peaks of MAPbI_3_ do not show a shift to larger angles, which would be supposed to happen in the case of K^+^ replacing the A‐site atoms. Consequently, it is deduced that K^+^ does not take the A‐site but, as reported, rather remains at grain boundaries and surfaces to passivate defects and is incorporated into KPbI_3_ crystallites under high‐KSCN‐concentration conditions.^[^ ^30^ ^]^

**Figure 2:**
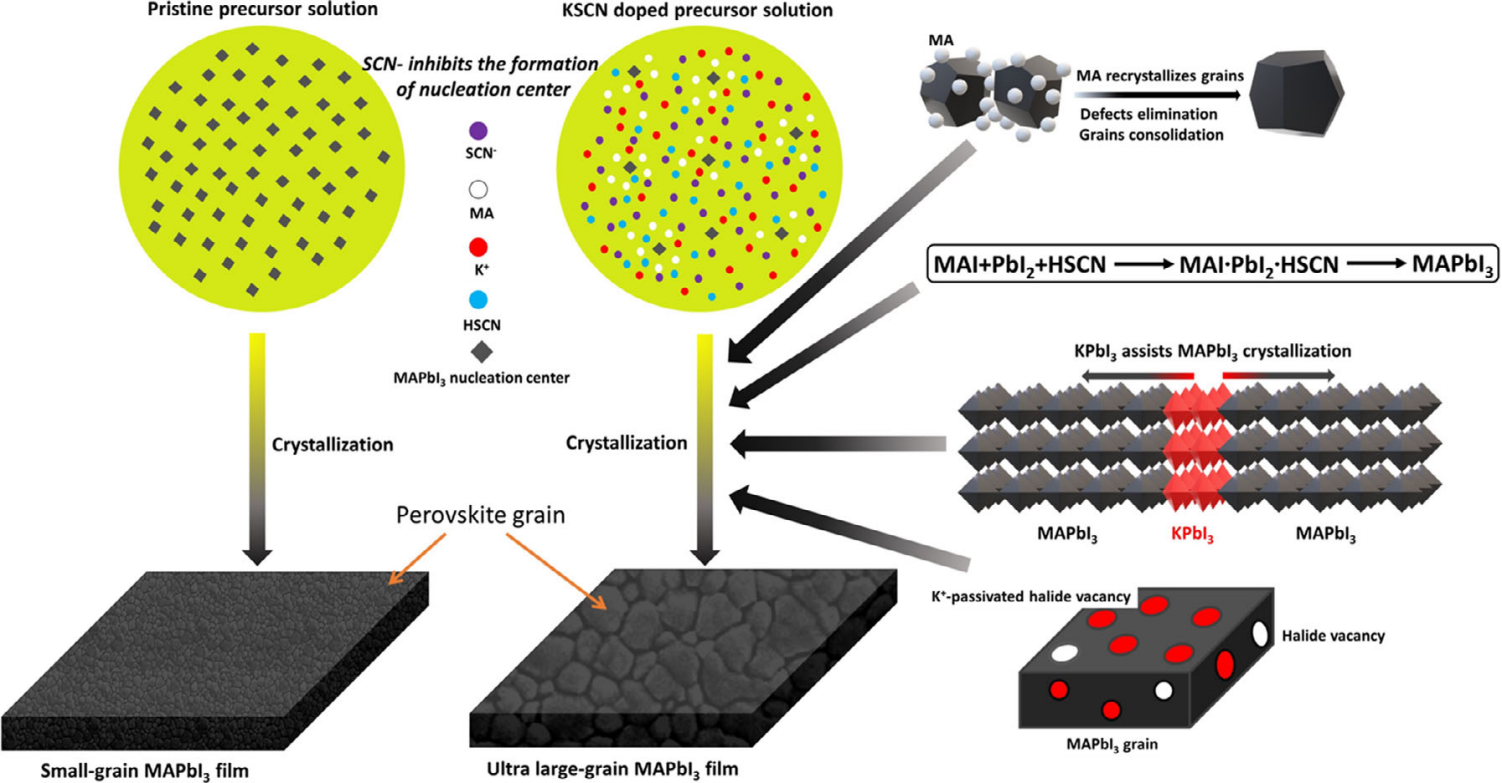
Sketch for the mechanism of forming ultra‐large‐grain MAPbI_3_ films and eliminating defects in films via KSCN additive.

The reactants can contribute to the formation of large crystal grains and the elimination of defects, as shown in **Figure** **2**. First, SCN^−^ (2.15 Å) has a radius close to that of I^−^ (2.20 Å), but a stronger electronegativity to form chemical bonds with cations, compared with I^−^, owing to its high polarity.^[^ ^24^, ^41^, ^42^ ^]^ Consequently, the left part (in blue circle) of Reaction 2 (Table S1, Supporting Information) is endothermic and $\Delta H_{\text{left}}$>0 ($\Delta H_{\text{left}}$ is the enthalpy change in the left part in the blue circle in Table S1, Supporting Information). Compared with the case of no SCN^−^, in which MAPbI_3_ nucleates as Reaction 4 (Table S1, Supporting Information), $\Delta H_{3}=\Delta H_{4}+\Delta H_{\text{left}}$>$\Delta H_{4}$. Here, $\Delta H_{3}$ and $\Delta H_{4}$ are the enthalpy changes in Reaction 3 and Reaction 4 in Table S1, Supporting Information. As a result, SCN‐ inhibits the formation of nucleation centers, resulting in larger grain growth. Second, MA^+^ is known to be incorporated into MAPbI_3_ grains, as shown in Reaction 3 (Table S1, Supporting Information).^[^ ^26^ ^]^ The recrystallization by MA^+^ promotes the elimination of defects and the consolidation of grains, enlarging the average grain size; traces can be seen in Figure S3b, Supporting Information. Third, HSCN, as shown in Figure S4, Supporting Information, is a Lewis base known to regulate the growth of lead iodide (PbI_2_) backbones of MAPbI_3_, resulting in grains of higher crystal quality.^[^ ^28^, ^29^, ^43^, ^44^ ^]^ Finally, KPbI_3_ can contribute to high‐quality MAPbI_3_ films because K^+^ can effectively passivate the halide vacancy, and the crystallized KPbI_3_ acts as the nucleation center for MAPbI_3_ for their common PbI_2_ backbones and the traces are shown in Figure S3b, Supporting Information.^[^ ^30^, ^31^ ^]^

**Figure 3:**
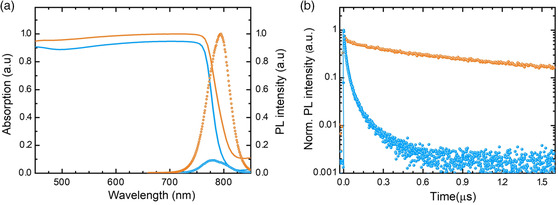
Optical characterizations. a) Absorptance, steady‐state PL, and b) time‐resolved photoluminescence (TRPL) spectra of pristine and KSCN‐modified MAPbI_3_ films.

Further characterization shows that the addition of KSCN improves the optoelectronic quality of slot‐die‐coated MAPbI_3_ films, which becomes comparable with that of perovskite single crystals. The top surface SEM images (Figure 1c) show that the average grain size increases from 0.23 μm (pristine MAPbI_3_) to 11.2 μm (KSCN‐modified MAPbI_3_). Thus, the ultra‐large average grain size reduces the number of grain boundaries; smaller grains usually cause instability and are detrimental to the perovskite films’ electronic quality. Moreover, these larger crystal grains enhance the optical absorption due to a red‐shifted bandgap of MAPbI_3_ thin films (**Figure** **3**a), as confirmed by photoluminescence (PL) (Figure S5a,b, Supporting Information) and the UV–vis absorption measurements (Figure S5e, Supporting Information), in line with earlier reports.^[^ ^44^, ^45^, ^46^ ^]^ Consequently, we find that in the KSCN‐modified films, the *J*_sc_ of solar cells is considerably improved due to enlarged grains. In addition, we find that the size of the grains in the transverse direction in KSCN‐modified films increases. Indeed, the cross‐sectional SEM image of the devices (Figure 1d) shows that the pristine films contain tiny grains. In contrast, KSCN‐modified films do not show in‐plane grain boundaries, improving charge‐carrier transport through perovskite films.

**Figure 4:**
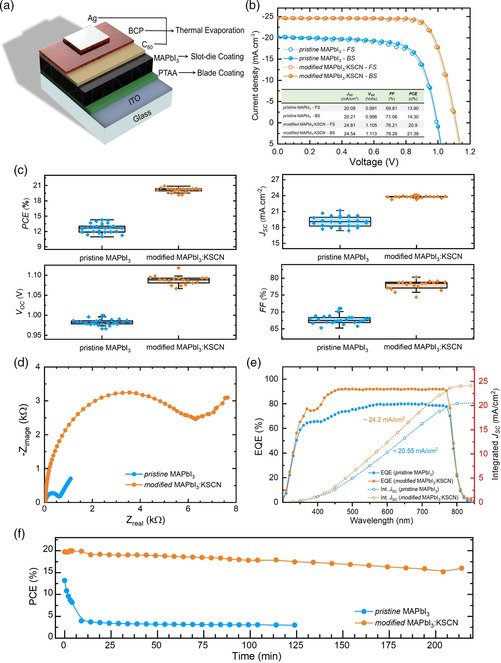
Device structure and solar cell characterization. a) Solar cell structure and the deposition method for individual layers. b) Champion *J–V* curves of slot‐die‐coated pristine and KSCN‐modified MAPbI_3_ PSCs. c) The statistical distribution of device parameters for PSCs using pristine and KSCN‐modified MAPbI_3_ alongside corresponding d) EIS measurement and e) EQE plots. f) Stability test results in N_2_ gas filled glovebox under continuous simulated AM1.5 solar illumination without intentional cooling, UV filter, and encapsulation.

Moreover, the intragrain defects in the films are markedly reduced as well. Figure 3a shows that KSCN‐modified films have a much higher PL intensity (Figure S5a, Supporting Information), up to 10.68 times higher than that of pristine MAPbI_3_ films, indicating that defects acting as nonradiative recombination centers have been significantly eliminated or passivated. This higher PL intensity for KSCN‐modified films is consistent with the electrical impedance spectroscopy (EIS) results (**Figure** **4**e), in its high‐frequency region; a larger radius of the semicircle implies a larger recombination resistance (*R*_rec_).^[^ ^47^ ^]^ Here, we find that the radius is enhanced 10.21 times, suggesting a reduction in charge recombination via the elimination of recombination centers. As crystal defects can act both as recombination and scattering centers of charge carriers, their reduction improves all carrier‐related parameters. The carrier lifetime was increased from 0.16 μs to a maximum of 1.89 μs, as observed from TRPL spectra (Figure 3b). Transient photovoltage (TPV) measurements (Figure S6d, Supporting Information) at 0.1 sun illumination showed 77.43 and 19.18 μs carrier lifetimes for the KSCN‐modified and pristine MAPbI_3_ PSCs, respectively, consistent with the TRPL results. The carrier mobility also improves from 2.35 ± 0.77 to 136.65±31.52 cm^2^ V^−1^ s^−1^ (Table S2, Supporting Information). According to the formula

(2) $$L=\sqrt{\frac{k_{B}T\tau \mu }{q}}$$

(in which *L* is the carrier diffusion length, *k*_B_ is the Boltzmann constant, *T* is the absolute temperature, *τ* is the carrier lifetime, *μ* is the carrier mobility, and *q* is the electron charge), the diffusion length improved from 0.99 ± 0.15 μm to 25.15 ± 3.55 μm, calculated using the carrier lifetime of TRPL. The data in Table S3, Supporting Information, showed that our polycrystalline films, fabricated by high‐speed slot‐die processing, have comparable charge‐carrier parameters as MAPbI_3_ single crystals.^[^ ^35^, ^36^ ^]^ The long carrier diffusion length is also manifested by the apparent peripheral carrier collection for PSCs built from these KSCN‐modified perovskites, a phenomenon earlier observed in single‐crystal PSCs.^[^ ^48^ ^]^. Such peripheral carrier collection mandates that all KSCN‐modified MAPbI_3_ PSCs’ performance is recalibrated by a calibration factor, as shown in Figure S6, Supporting Information. The elimination of trap defects also reduces the surface potential distribution, as evidenced by Kelvin‐probe force microscopy (KPFM) results (Figure S7, Supporting Information). This reduction causes less distortion of the built‐in electric field around the interfaces between the perovskite and hole or electron transport layers (HTL or ETL) in PSCs and is beneficial for charge‐carrier transport. Transient photocurrent (TPC) decay measurements (Figure S6e, Supporting Information) support this claim, where the photocurrent decay time lowers from 892 ns (pristine MAPbI_3_ PSC) to 806 ns (KSCN‐modified MAPbI_3_ PSC). As the charge‐carrier transport and collection enhance, the micrometer‐thick MAPbI_3_ films enable improved performance of PSCs. The micrometer‐thick PSCs exhibited better near‐band‐edge external quantum efficiency (EQE) than conventional PSCs with ≈500 nm absorber layers (Figure S6a, Supporting Information), similar to that of single‐crystal PSC,^[^ ^35^ ^]^ which also results in an enhanced *J*_sc_.

Overall, the excellent properties of the KSCN‐modified films combined with the micrometer‐thick perovskite absorbers improved device performance and reproducibility. Figure 4c consolidates the device statistics for control and KSCN‐modified PSCs. The average PCE for control PSCs built from pristine MAPbI_3_ films is only 12.60 ± 1.70%, with an average *J*_sc_  = 19.06 ± 2.13 mA cm^−2^, *V*_oc_  = 0.982 ± 0.017 V, and FF = 67.26 ± 3.80%. For the PSCs using KSCN‐modified films, the average PCE increases ≈1.5 times to 20.14 ± 0.75%, with an average *J*_sc_  = 23.78 ± 0.35 mA cm^−2^, *V*_oc_  = 1.087 ± 0.031 V, and FF = 77.93 ± 2.35%. These results underline the slot‐die method's promise as the average PCE for our KSCN‐modified PSCs is comparable with the results by lab‐scale deposition methods.^[^ ^49^, ^50^ ^]^ Figure 4b shows the *J–V* curves for the best cells. Due to KSCN modification, all main PV metrics improve. The best cell fabricated by pristine MAPbI_3_ films gave a PCE of 14.30%, with a *J*_sc_ = 20.21 mA cm^−2^, *V*_oc_ = 0.996 V, and FF = 71.06%. The best KSCN‐modified MAPbI_3_ PSC shows *J*_sc_ = 24.54 mA cm^−2^, *V*_oc_ = 1.113 V, and FF = 78.26%, resulting in a PCE of 21.38%. Figure 4e shows the EQE from 300 to 900 nm. The KSCN‐modified MAPbI_3_ films exhibit much higher EQE values than the pristine MAPbI_3_ films. Integration of the EQE over the AM1.5 G solar spectrum yields photocurrents of 24.20 and 20.55 mA cm^−2^ for the KSCN‐modified and pristine reference devices, respectively, in excellent agreement with the measured *J*_sc_ values. This confirms the accuracy of *J*_sc_ values measured by *J–V*. In addition, the slight redshift in EQE, shown in Figure S6b, Supporting Information, corresponds to the bandgap energy lowering, as discussed earlier.

*I–V* measurements under different scan directions were applied to examine the hysteresis of our cells. The median of the HF, in which

(3) $$\text{HF}=\frac{\left|{\text{PCE}}_{\text{rev}}-{\text{PCE}}_{\text{fw}}\right|}{\text{MAX}\left({\text{PCE}}_{\text{rev}},{\text{PCE}}_{\text{fw}}\right)}\times 100\%$$

reduces from 4.75% (pristine MAPbI_3_ PSCs) to 0.59% (KSCN‐modified PSCs), and several PSCs even had no hysteresis (HF is 0; Figure S6e, Supporting Information). The enlarged perovskite crystal size and improved crystalline quality eliminate hysteresis, likely reducing the overall bulk defect density and suppressing charge trapping. The improved bulk quality is confirmed by the reduced density of states (DOS) distributions derived from admittance spectroscopy (Figure S6h, Supporting Information). A preliminary stability investigation shows that KSCN modification improves the operational stability of devices. Figure 4f compares the stability of PSCs based on pristine and KSCN‐modified MAPbI_3_ films under continuous simulated AM1.5 G solar illumination without cooling systems, UV filter, or encapsulation. Within 9 min, the PCE of the pristine MAPbI_3_ PSC drops from 13.19% to 3.97%. In contrast, for KSCN‐modified MAPbI_3_ PSCs, the PCE decreases from 19.74% to 16% after 214 min. Considering the known thermal and radiative instability of MAPbI_3_, this result is impressive. The vast differences in device performance indicate that the KSCN modification is a simple and effective approach to enhance the charge‐carrier parameters and stability of perovskite materials, likely also applicable using other deposition methods than slot‐die coating.

Finally, Figure S7g, Supporting Information, shows the *J–V* curves of nine pixels of PSCs located along the slot‐die axis, covering a length of 7.5 cm. The variance of their PCE extracted from the nine curves is as little as 0.0023. This narrow PCE dsitribution attests to the uniformity of the slot‐died perovskite films over the large area. Note that as shown in Figure 4a, all PSCs fabrication layers use scalable methods, including blade coating, slot‐die coating, and thermal evaporation.

## 3 Conclusion

We demonstrated KSCN‐assisted slot‐die‐coated MAPbI_3_ films with ≈11 μm large grains and 25.15 ± 3.55 μm charge‐carrier diffusion length. To take the advantage of long charge‐carrier diffusion length and the better band‐edge absorption in micrometer‐thick PSCs, we then fabricated high‐performance p–i–n planar MAPbI_3_ PSCs (best PCE = 21.38% and average PCE = 20.14%) with negligible hysteresis (average HF = 0.59%) and improved stability. The entire device stack of the PSC was fabricated by scalable methods that pave the way toward advancements in high‐throughput deposition techniques. These results bring the quality of slot‐die‐prepared perovskites to a comparable level with that of other lab methods. Moreover, our results encourage adopting KSCN treatment to other perovskite deposition techniques, including those required to fabricate efficient perovskite/silicon tandem solar cells for ultrahigh performance.^[^ ^51^, ^52^, ^53^, ^54^ ^]^

## 4 Experimental Section

4.1

4.1.1

##### Materials

KSCN (>99%), bathocuproine (BCP, 99.99%), and ACN (ACN ≥ 99.9%) were obtained from Sigma‐Aldrich. PbI_2_ (99.999%) was purchased from Alfa Aesar. Methylammonium iodide (MAI) was procured from GreatCell Solar (Australia). MA methanol solution (9.8 mol L^−1^) was purchased from Tokyo Chemical Industry. All materials were used as received without any further purification. The PTAA was purchased from Xi'an Polymer Light Technology.

##### Methods—Solar Cell Fabrication

Patterned indium tin oxide (ITO)/glass substrates were sequentially cleaned with soap, deionized water, acetone, and isopropanol under ultrasonication. PTAA solution (5 mg mL^−1^) was blade‐coated on patterned ITO using a 10 mm s^−1^ blade‐coating speed, and the coated films are annealed at 100 °C for 10 min. MAPbI_3_ solution (1 m) was prepared by dissolving MAI and PbI_2_ (molar ratio MAI:PbI_2_ = 1:1) into MA methanol solution and ACN (volume ratio MA methanol solution: ACN = 2:3). *x*M KSCN‐doped MAPbI_3_ solutions are prepared by dissolving KSCN powders into the MAPbI_3_ solution with *x*:1(KSCN:MAPbI_3_) molar ratio. The solution is slot‐died onto the 70 °C PTAA films with 0.10 mL s^−1^ solution supply rate, 15 mm s^−1^ slot‐die rate. The gaps between slot‐die head and substrate were 0.45 and 0.30 mm for KSCN‐modified MAPbI_3_ films and pristine MAPbI_3_ films, respectively. The slot‐died films are dried on a hot plate at 85 °C for 20 min. Then the 8 nm BCP, 35 nm C_60_, and 150 nm silver are thermally evaporated on deposited perovskite films subsequently.

##### Methods—Characterization

The XRD spectra of the prepared films were measured using a Bruker D2 Advance diffractometer (Bruker, PHASER) with an X‐ray tube (Cu Kα, *λ* = 1.5406 Å). XPS was conducted with an OmicronScienta equipment equipped with a XM1000 monochromatic Al Kα X‐ray source (1486.6 eV) and a Sphera II EAC125 hemispherical analyzer. The spectra were recorded with 15–50 eV constant pass energy at ground potential with the sample in electrical contact with the analyzer. Binding energy calibration was made to an Ag foil. Spectra were fit and analyzed in CasaXPS. SEM images were taken with a ZEISS MERLIN Field Emission Scanning Electron Microscope. AFM and KPFM images were measured by the machine from Veeco Metrology Group. Transmittance (*T*) and reflectance (*R*) are measured by PerkinElmer Lambda 950 UV–vis–NIR spectrophotometer, and the absorption (*A*) is calculated by *A* = 100–(*T* + *R*). Hall effect is performed on Lake Shore Hall effect system at room temperature and dark conditions. Steady‐state PL was measured by Fluoroma‐4, Horiba Scientific with an excitation wavelength of 500 nm from a monochromated xenon light source, and the same machine measured TRPL with 633 nm pulsed laser. The EQE was measured using a QEX10 Spectral Response Measurement System (PV Measurements, Inc.), calibrated against a reference silicon photodiode. Current–voltage characterizations are tested with a Keithley 2400 Sourcemeter and a Class AAA solar simulator equipped with a 150 W xenon arc lamp (Sun 3000, Abet Technologies) under AM1.5 G illumination, and the light intensity was calibrated with a certified reference cell (RERA Solutions, calibrated at Radboud University Nijmegen). The voltage was swept with a speed of 50 mV s^−1^ for both forward and reverse scans. The area of copper mask for solar cells testing is 0.089 cm^2^confirmed by an optical microscope. The TPV, TPC, EIS, and admittance were tested by PAIOS: Platform for All‐In‐One Characterization of Solar Cells. EIS was tested with the sweep frequency from 10 MHz to 300 Hz with the steps of 100. The sweep offset voltage is 0 V. The offset light intensity is 100%, and the amplitude is 25 mV. The DOS (distribution) derived from admittance spectroscopy is calculated by the formula $Nt(E_{\omega })=-\frac{V_{\text{bi}}}{qW}\frac{dC}{d\omega }\frac{\omega }{k_{B}T}$, where *W*, *V*_bi_, *q*, *k*_B_, *T*, *ω*, and *C* are the depletion width, build‐in potential, Boltzmann constant, electron charge, temperature, angular frequency, and specific capacitance. The demarcation energy $E_{\omega }=k_{B}T\ln(\frac{\omega_{0}}{\omega })$, where *ω*_0_ is the attempt‐to‐escape angular frequency $\omega_{0}=2\pi \upsilon_{0}T^{2}$ and *υ*_0_ is the temperature‐independent attempt‐to‐. We assume *ω*_0_ = 1 × 10^10^, *W* is 1000 nm, *T* is the room temperature, and *V*_bi_ = *V*_oc_ of the corresponding devices approximately as in the reported results.^[^ ^55^, ^56^ ^]^

## Conflict of Interest

The authors declare no conflict of interest.

## Data Availability Statement

Research data are not shared.

## Supporting information

Supplementary Material
