# Supplementary material for: Potassium Thiocyanate‐Assisted Enhancement of Slot‐Die‐Coated Perovskite Films for High‐Performance Solar Cells
Source: Small Sci. 2021 Feb 7;1(5):2000044. doi: 10.1002/smsc.202000044 (PMC11935903; doi:10.1002/smsc.202000044)
Supplement: Supplementary file 1 — Supplementary Material [file SMSC-1-2000044-s001.docx]

Supporting Information

**Potassium Thiocyanate-Assisted Enhancement of Slot-Die-Coated Perovskite Films for High-Performance Solar Cells**

*Fuzong Xu,^a^ Jiang Liu,^a^ Anand S. Subbiah,^a^ Wenzhu Liu,^a^ Jingxuan Kang,^a^ George T. Harrison, ^a^ Xinbo Yang,^a^ Furkan H. Isikgor,^a^ Erkan Aydin,^a^ Michele De Bastiani,^a^ and Stefaan De Wolf^*a^*

**This PDF file includes:**

Peripheral collection and calibration factor

Tables. S1 to S3

Figures. S1 to S7

**Peripheral collection and calibration factor:**

As reported, the long charge carrier diffusion length causes photogenerated charge carriers around active area being effectively collected for additional current. It is called peripheral collection and is common in small-area single-crystal solar cells.

In our work, KSCN modified MAPbI_3_ films have single-crystal-level charge carrier diffusion length, and KSCN modified PSCs show the peripheral collection. As shown in Fig.S7(C), Fig.3(A) and (B), the mismatch between the J-V *J_sc_* and EQE calculated *J_sc_* appears in the best KSCN modified MAPbI_3_ PSC which has 25.15 μm diffusion length but not in the pristine MAPbI_3_ PSC which has 0.99 μm diffusion length. Besides, testing with a mask, the best KSCN modified MAPbI_3_ PSC has a 24.54 mA/cm^2^ *J_sc_* which almost equals to the *J_sc_* by EQE. Using a normal KSCN modified MAPbI_3_ PSC to double check that. As shown in Fig.S7(C), testing with a mask and without a mask, the difference of J-V curves is obvious as well, and the ratio between the PCEs in the two testing conditions of the normal and the best PSCs are 1.045 and 1.040 respectively. They are almost the same, and testing with mask is an effective and reliable way to get the accurate J-V results. All the phenomena show that, if light incidents on only the active area but the area around active area, the PSC show very high *J_sc_* which is not corresponds to the EQE, which is corresponding to the reported peripheral collection. However, if light just incidents on active area, the PSC has a lower *J_sc_* but corresponds to the EQE.

Consequently, according to the devices tested with a mask and without a mask as shown in Fig.S7(c), all parameters of KSCN-modified PSCs are calibrated as the following formula:

$${Jsc}_{calibrated}={Jsc}_{without mask}\times0.945$$

$${Voc}_{calibrated}={Voc}_{without mask}\times0.996$$

$${FF}_{calibrated}={FF}_{without mask}\times1.016$$

$${PCE}_{calibrated}={PCE}_{without mask}\times0.956$$

, in which the calibration factors (0.945 for J_sc_, 0.996 for V_oc_, 1.016 for FF, and 0.956 for PCE) are calculated from the parameters difference in devices tested with a mask and without a mask. The parameters of devices shown in the paper are calibrated or tested with a mask.

**Table.S1** The chemical reactions in this work.


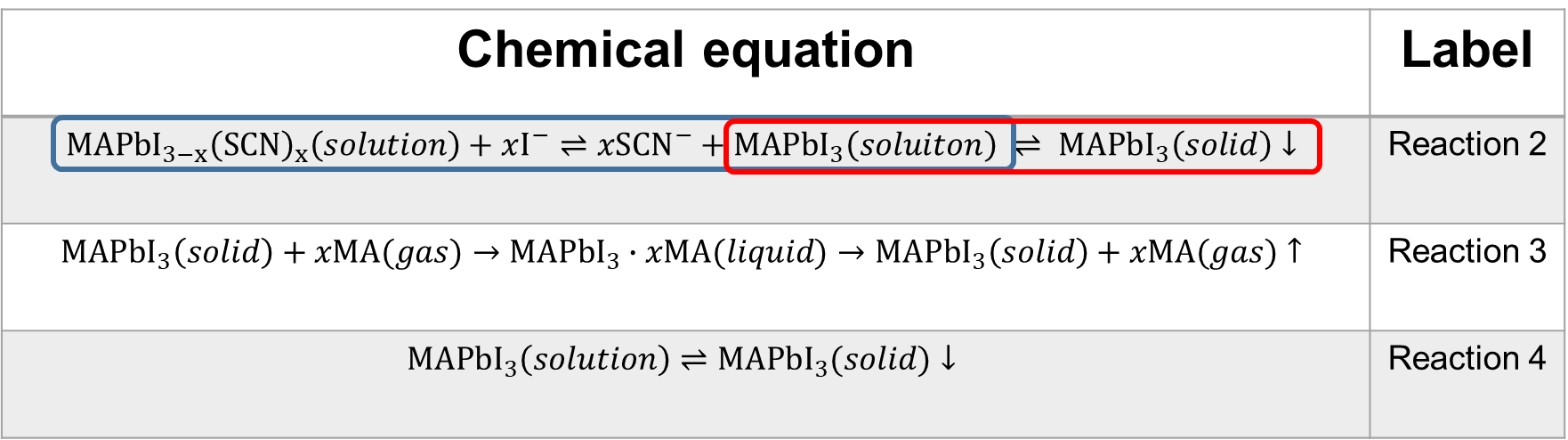


**Table.S2** The Hall effect results in this work.


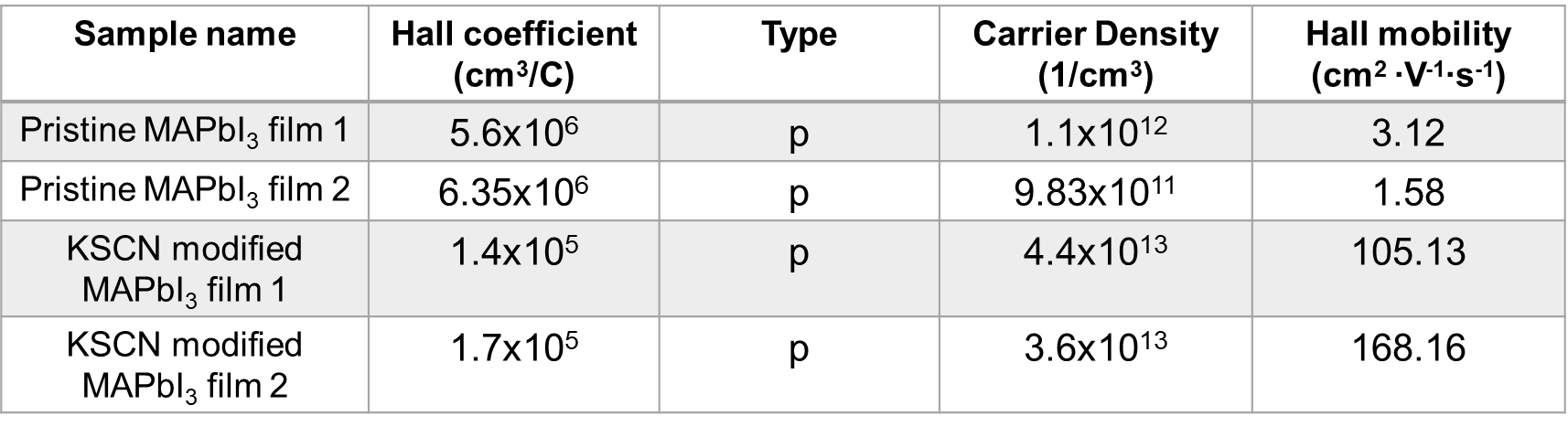


**Table.S3** Charge carrier parameters of excellent MAPbI_3_ single crystals in *(30,31)* by different testing methods and our films.


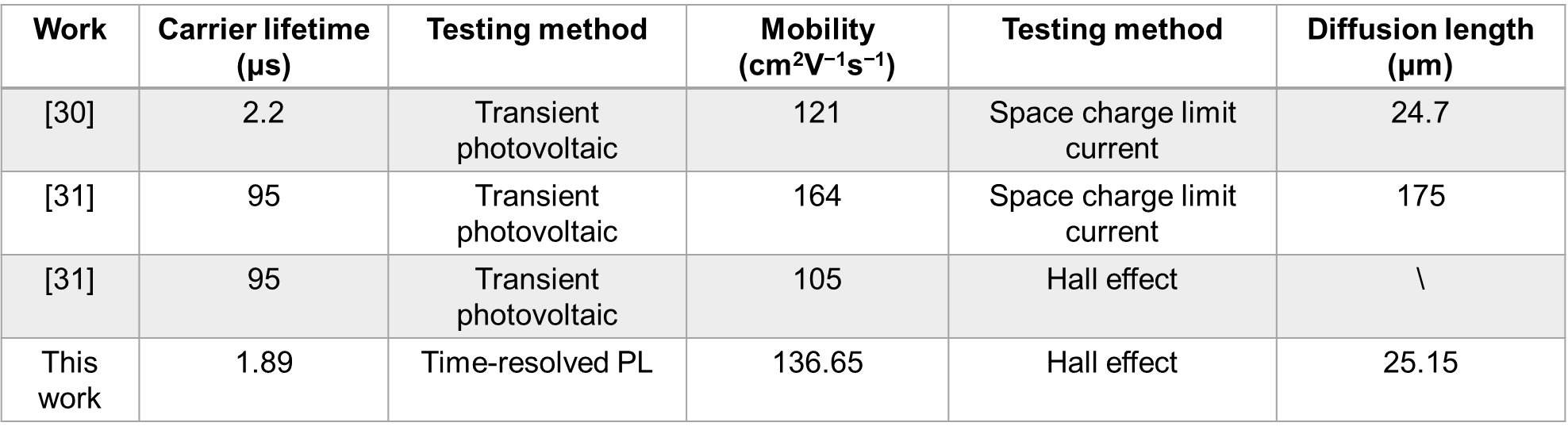


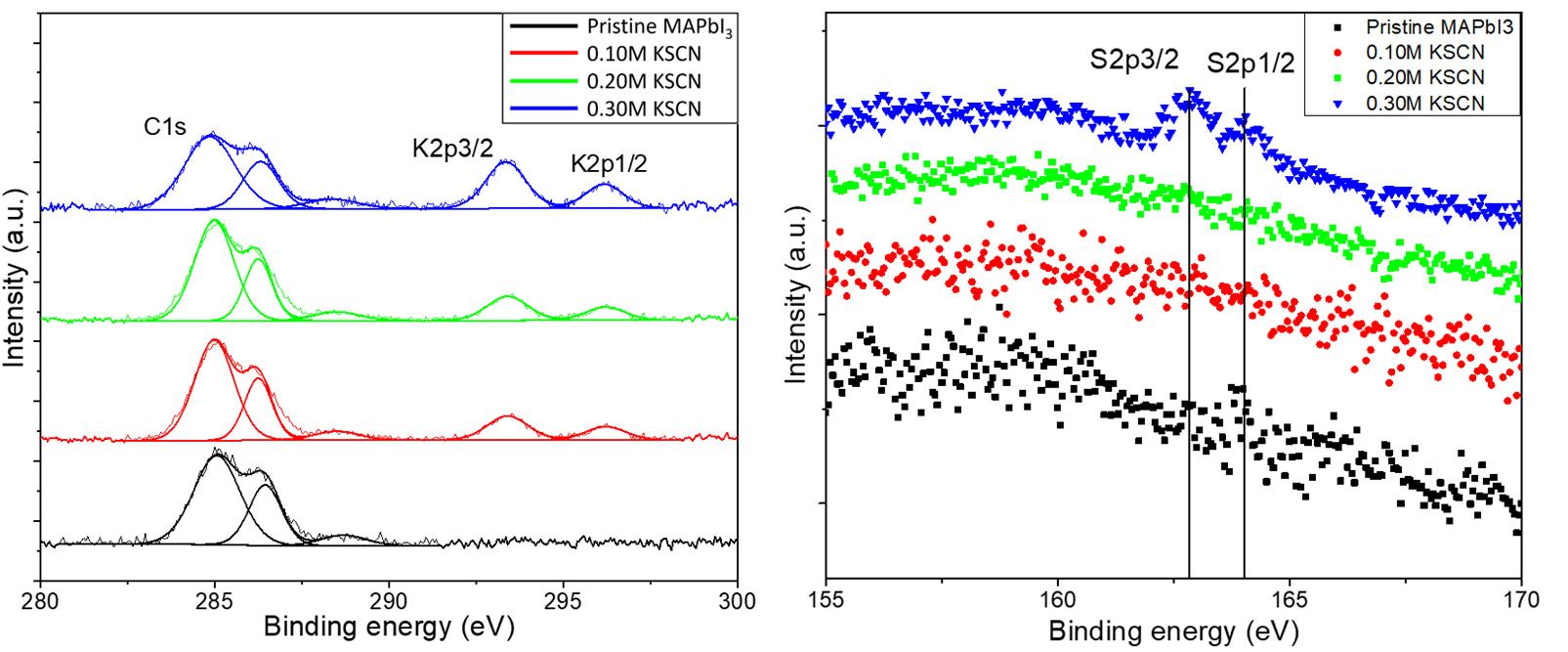


**Figure. S1.** XPS spectra of C1s and K 2p (left), S 2p (right) perovskite films by MAPbI_3_ precursor solutions doped by 0M, 0.10M, 0.20M and 0.30M KSCN


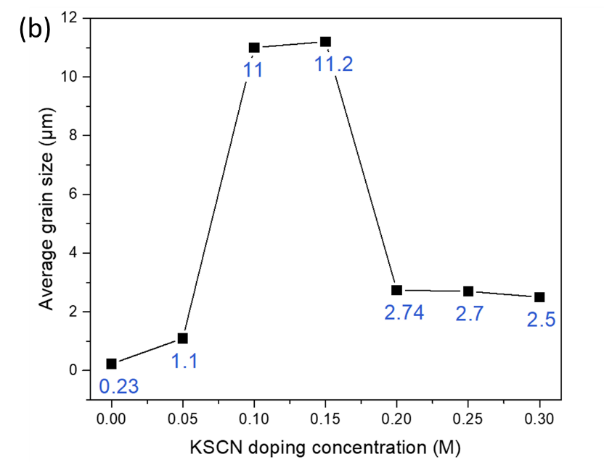

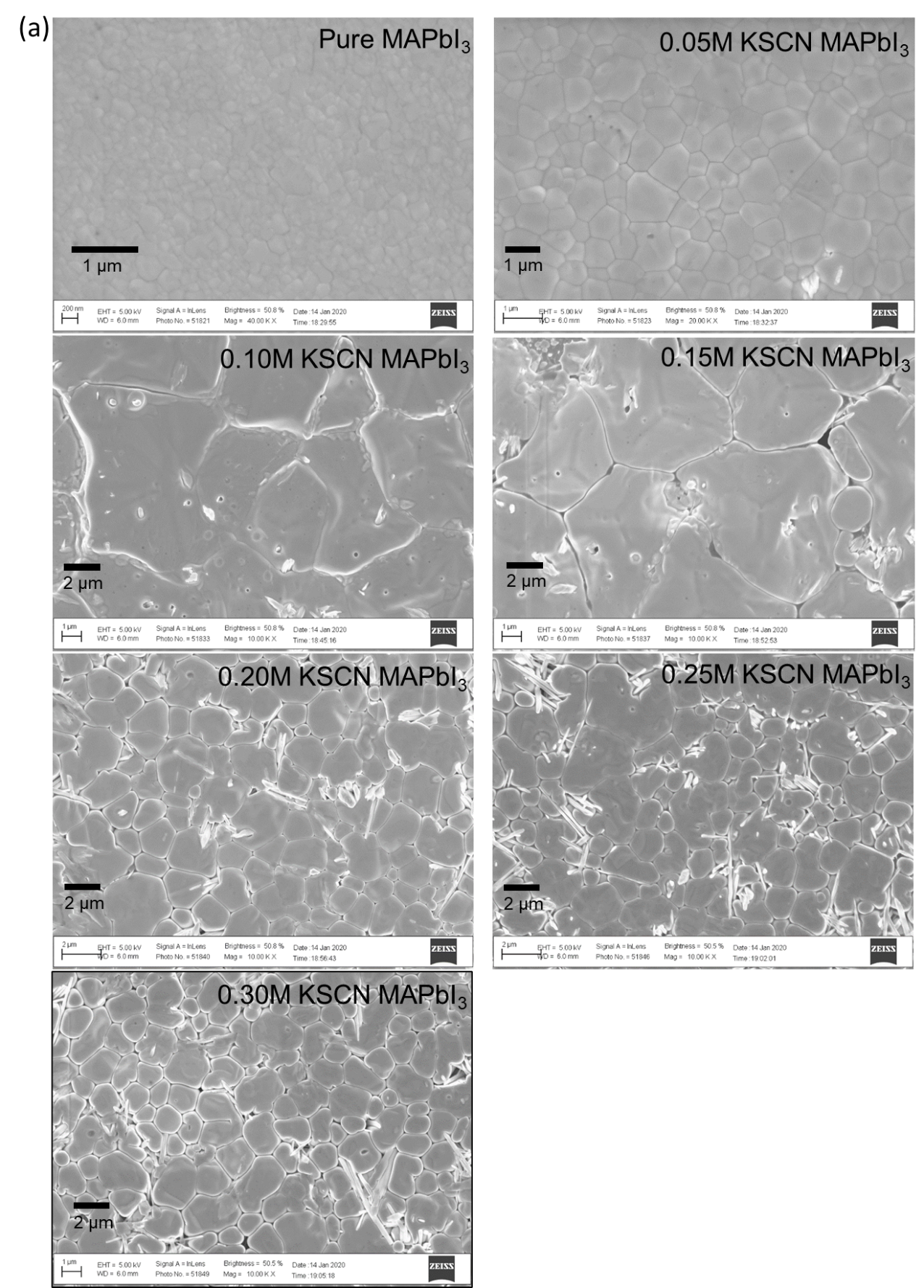


**Figure. S2. Scanning electron microscope (SEM).** Surface SEMs **(a)** and average grain size comparison **(b)** of perovskite films deposited by MAPbI_3_ precursor solutions doped by KSCN in the concentration of from 0 to 0.30M with a 0.05M step on PTAA.


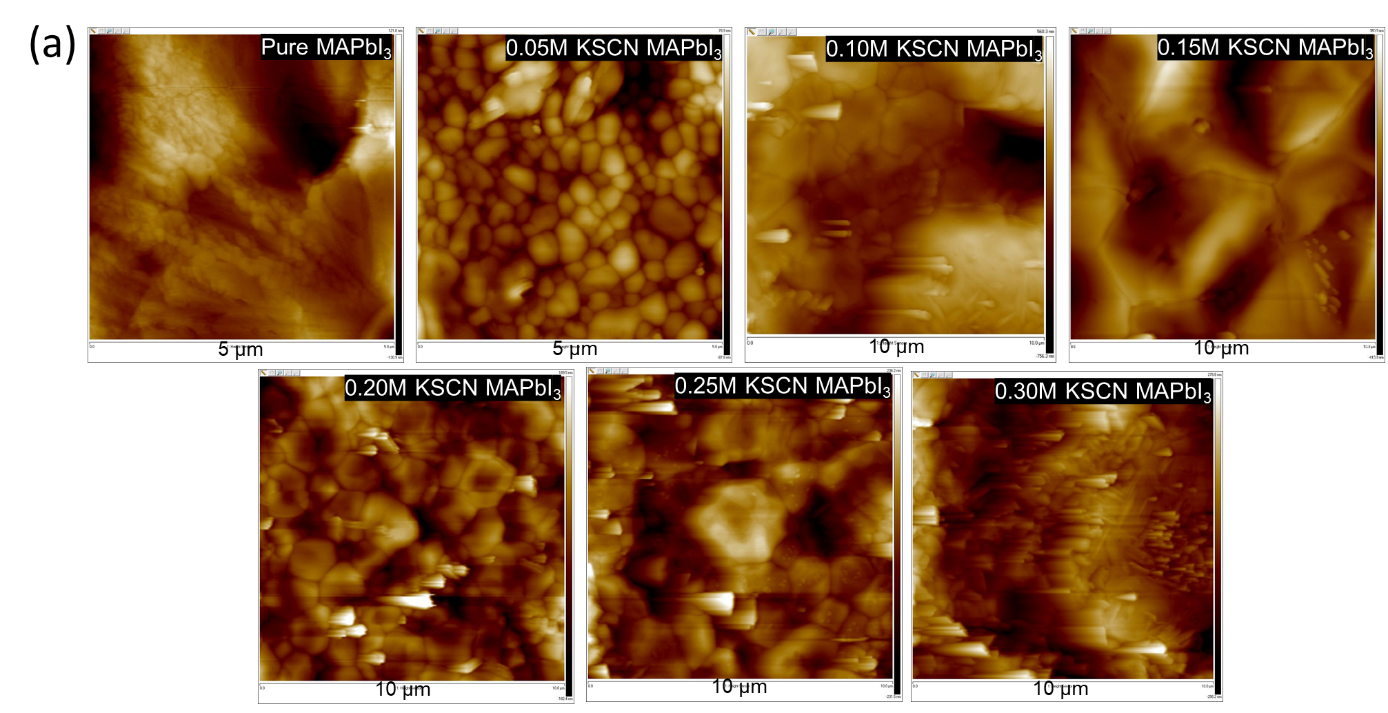


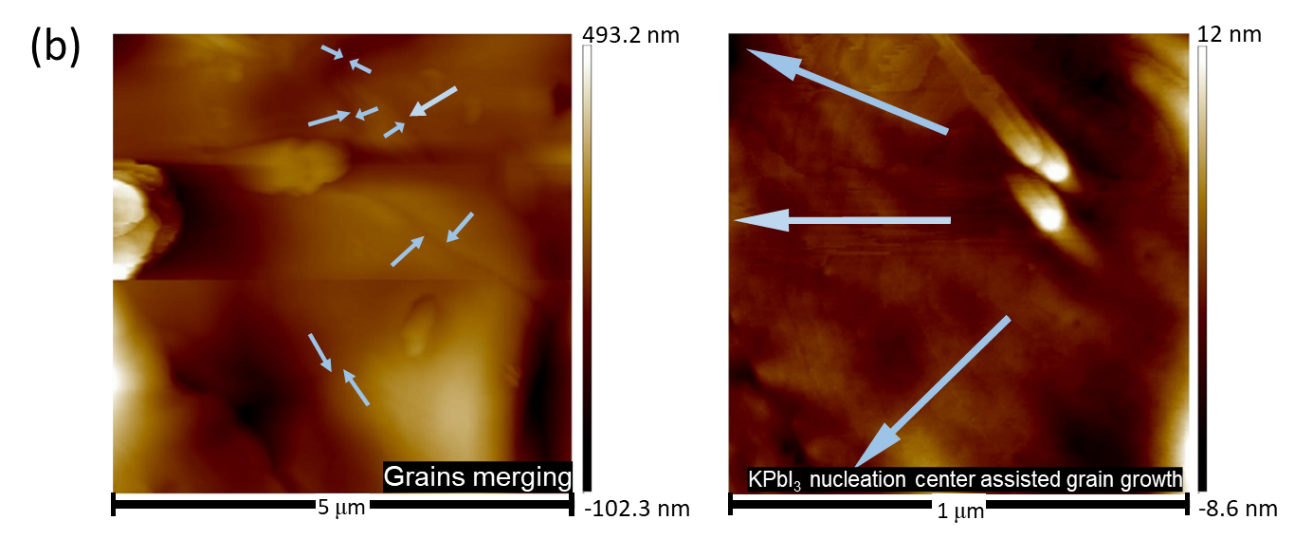


**Figure. S3. Atomic force microscope images.** **(a)**Surface AFM images of perovskite films deposited by MAPbI_3_ precursor solutions doped by KSCN in the concentration of from 0 to 0.30M with a 0.05M step on glass. **(b)** the traces of grains merging process and KPbI_3_ nucleation center assisted grain growth process.


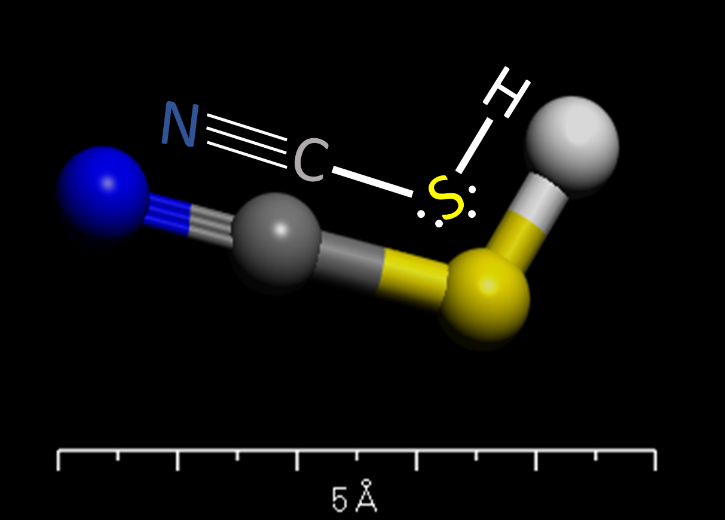


**Figure. S4.** Molecular structure of HSCN.


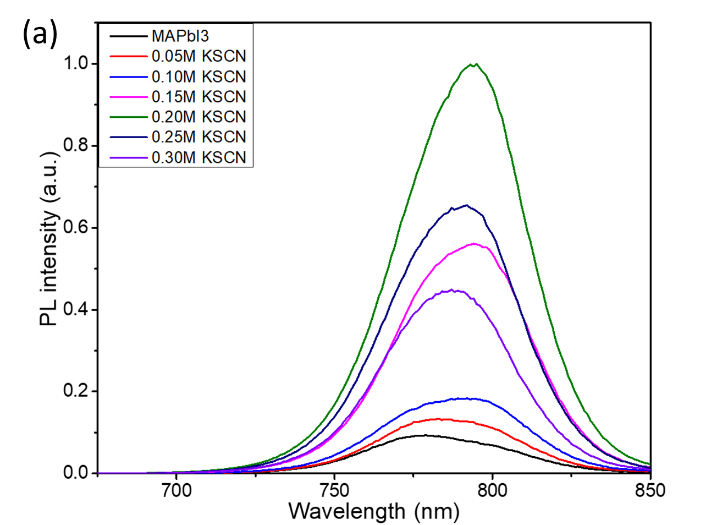

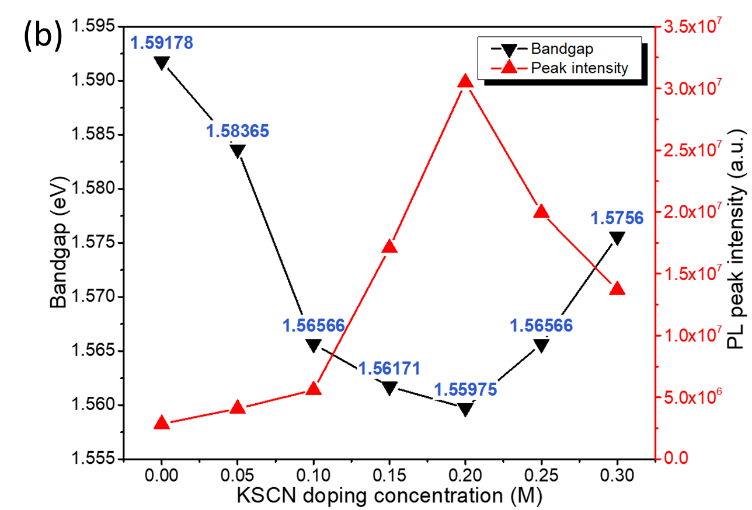


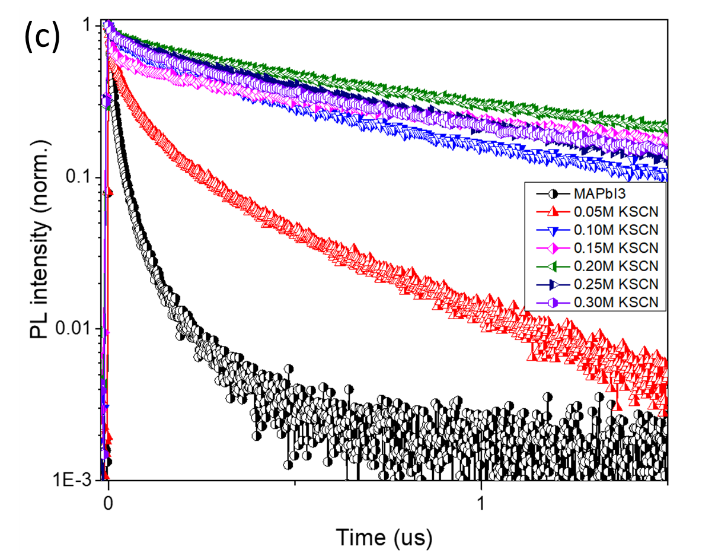

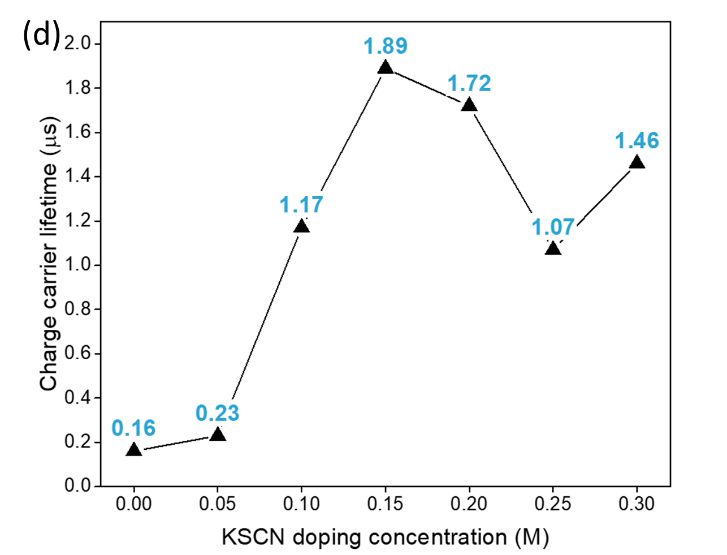


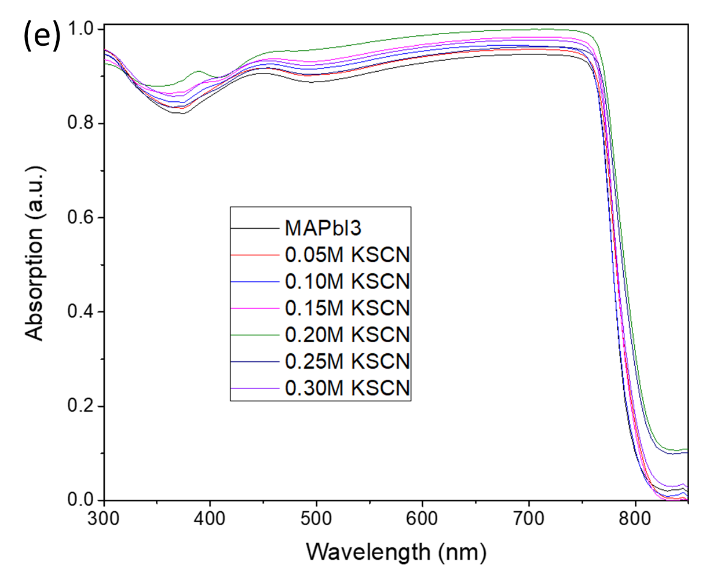


**Figure. S5.** **Optical characterizations.** **(a)**Photoluminescence (PL) spectrum, **(b)**results of bandgap and PL intensity based on **(a)**, **(c)**time-resolved photoluminescence(TRPL) spectrum, **(d)** results of carrier lifetime based on **(c)**, and **(e)**absorption spectrum of perovskite films deposited by MAPbI_3_ precursor solutions doped by KSCN in the concentration of from 0 to 0.30M with a 0.05M step.


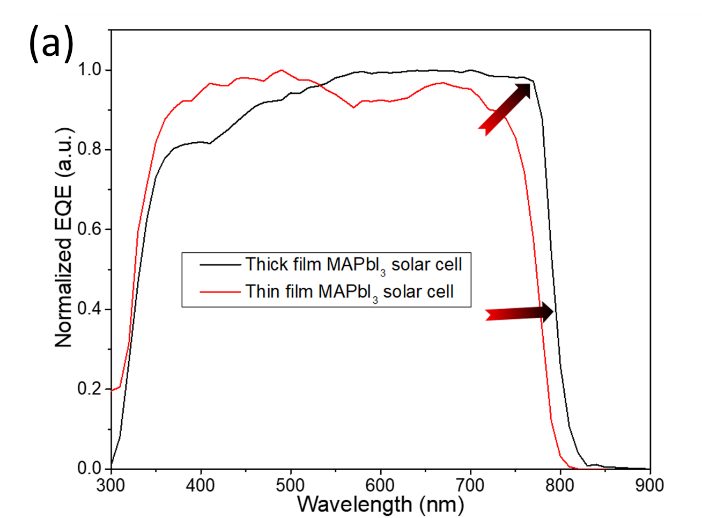

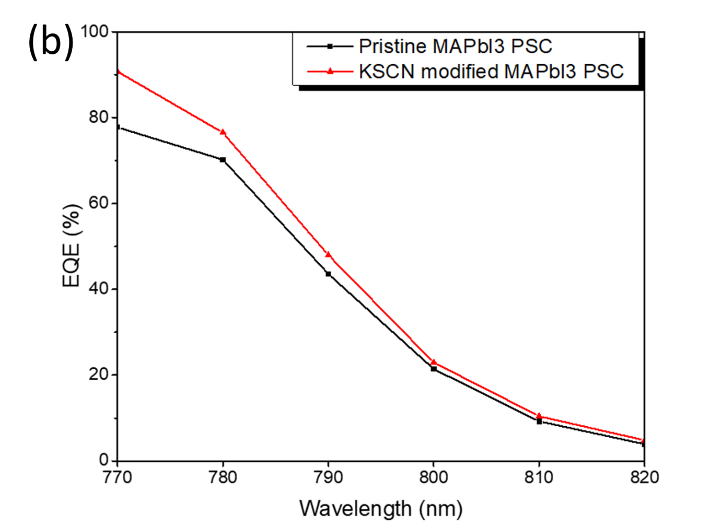


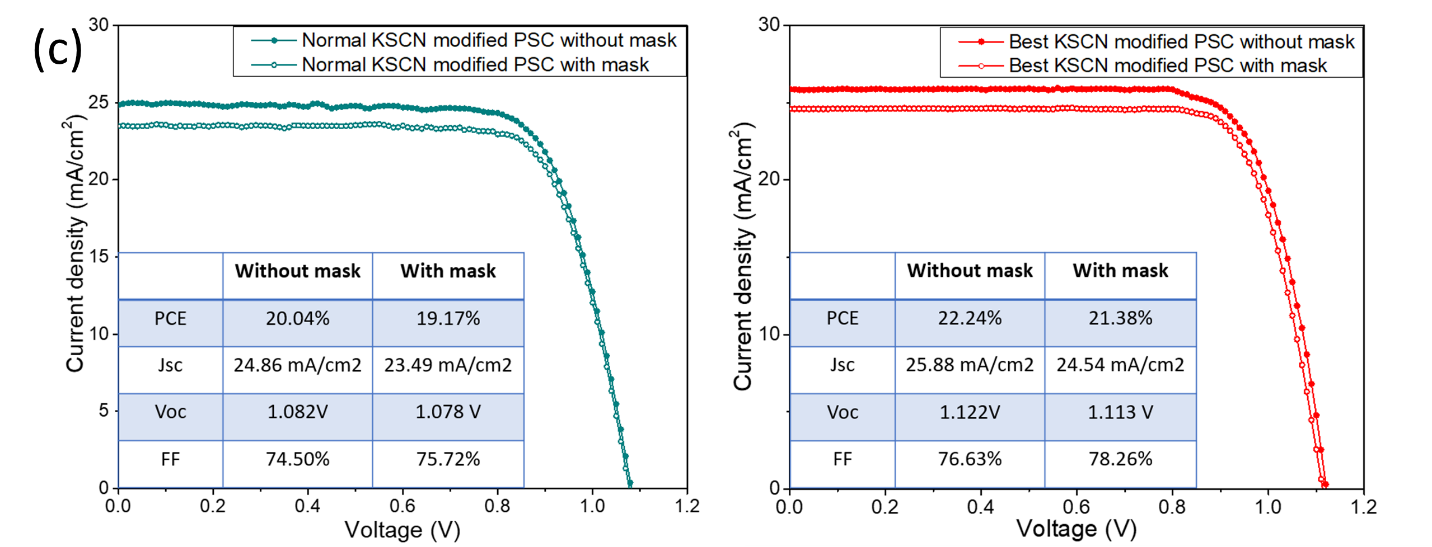


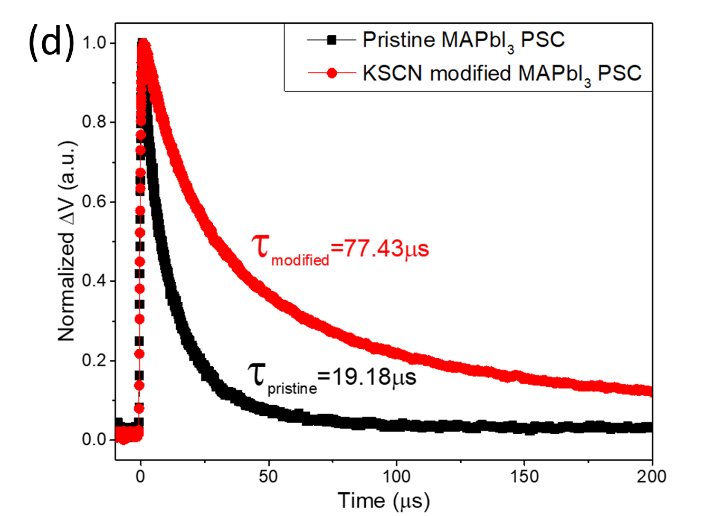

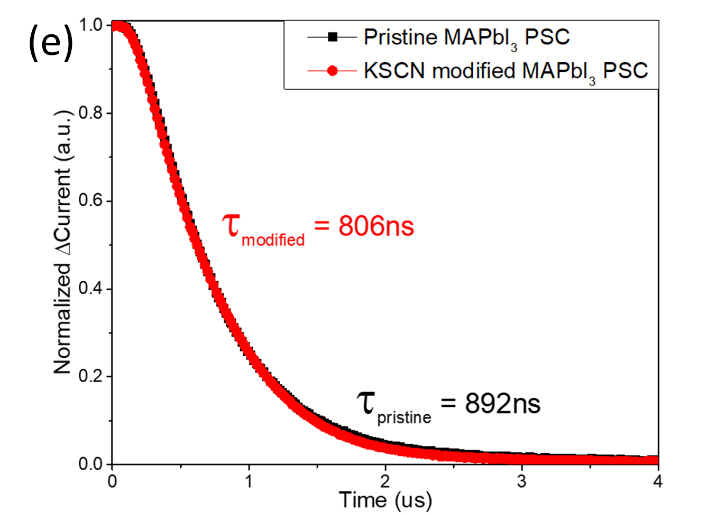


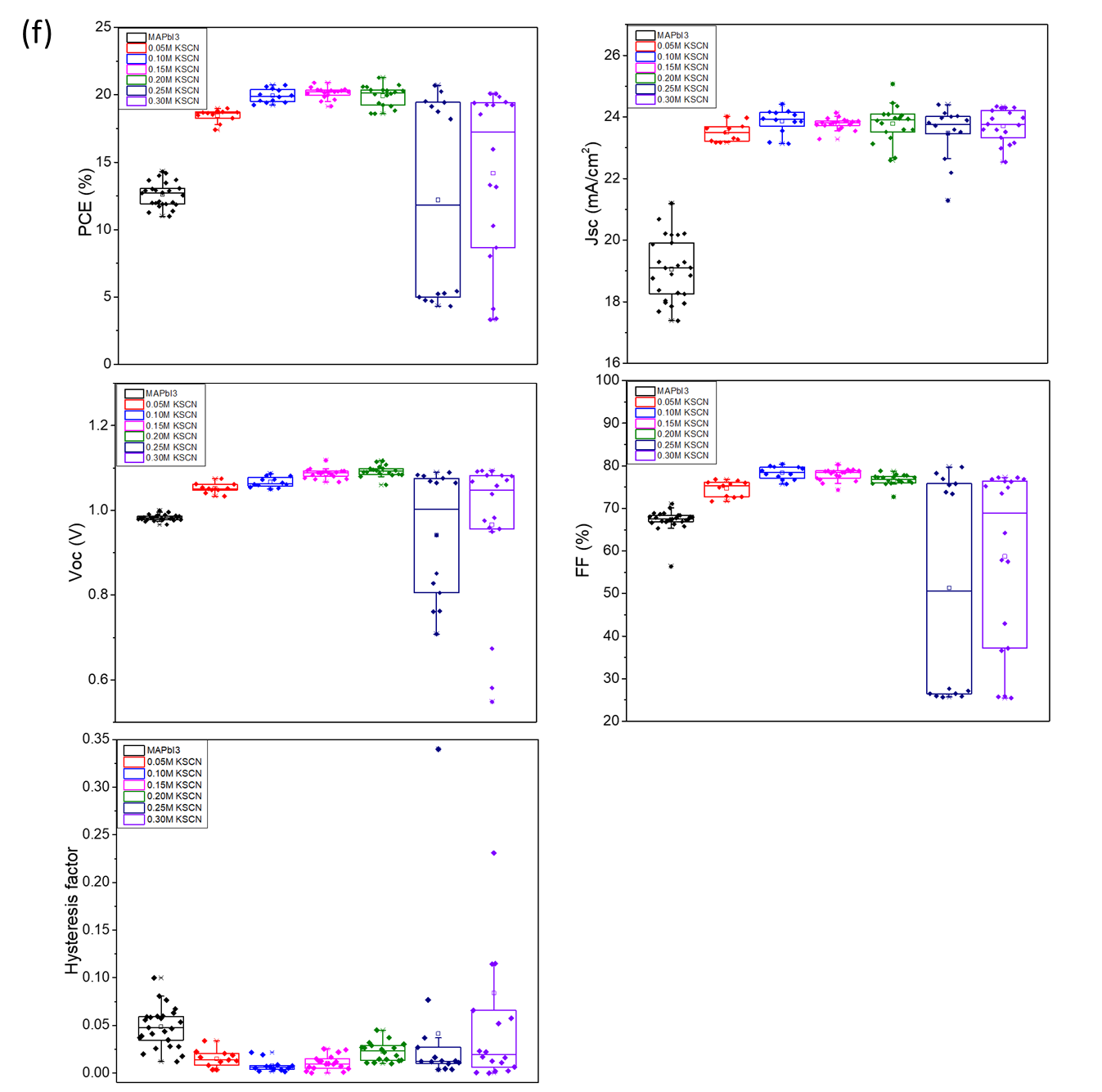


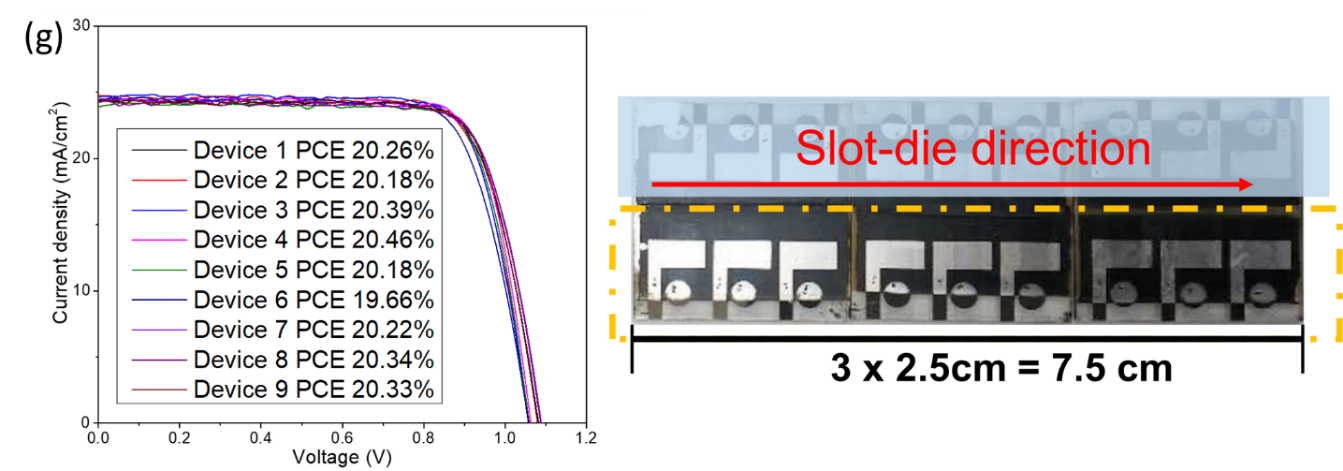


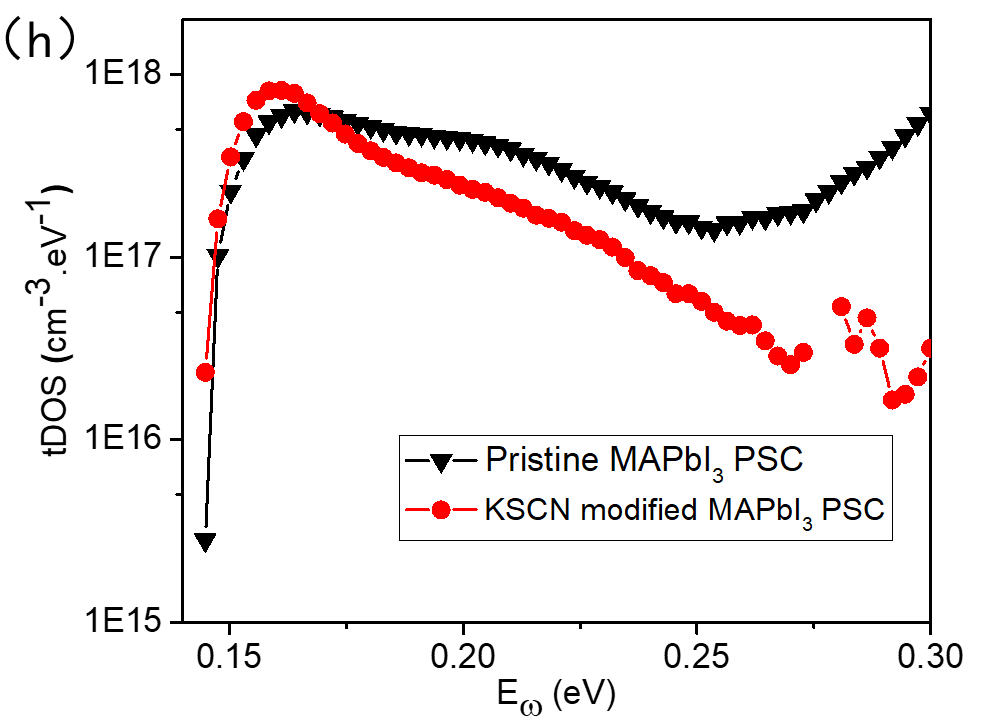


**Figure. S6. Devices characterizations. (a)** Normalized EQE of solar cells on thin and micro-thick pristine MAPbI_3_ films. **(b)** The EQE of pristine MAPbI_3_ PSC and KSCN modified MAPbI_3_ PSC from 770nm to 820nm in Fig.3C. **(c)** J-V curves for the best (red lines) and a normal (green lines) PSCs on KSCN modified MAPbI_3_ films with and without mask. The transient photovoltaic**(d)** and transient current**(e)** decay of pristine and KSCN modified MAPbI_3_ PSC**. (f)** The J-V parameters of PSCs on perovskite films deposited by MAPbI_3_ precursor solutions in the concentration of from 0 to 0.30M with a 0.05M step. **(g)** 9 PSCs on the 7.5cm KSCN modified MAPbI_3_ films and the J-V curves of them. (h) Density of states (DOS) distribution derived from admittance spesctroscopy.


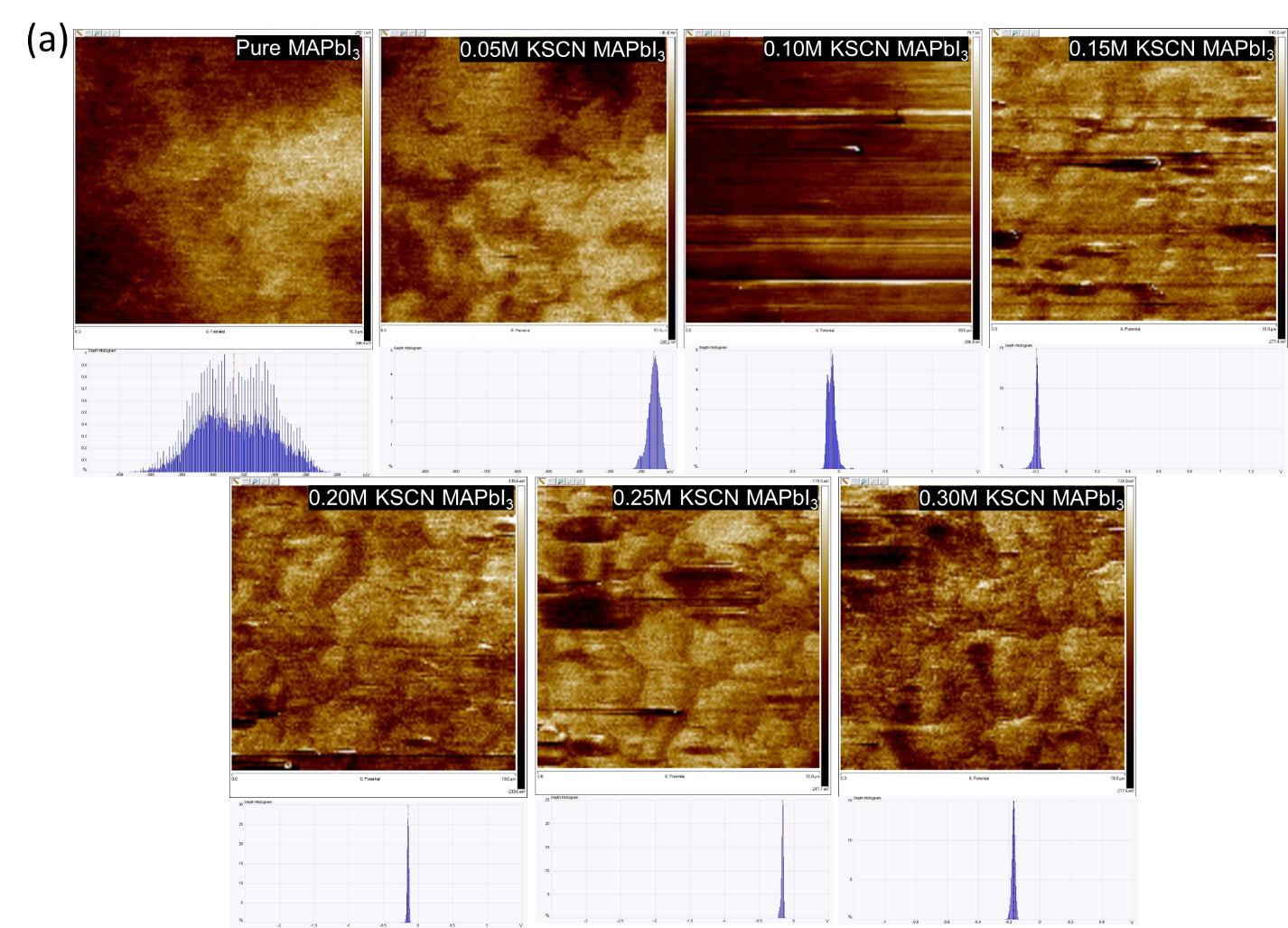


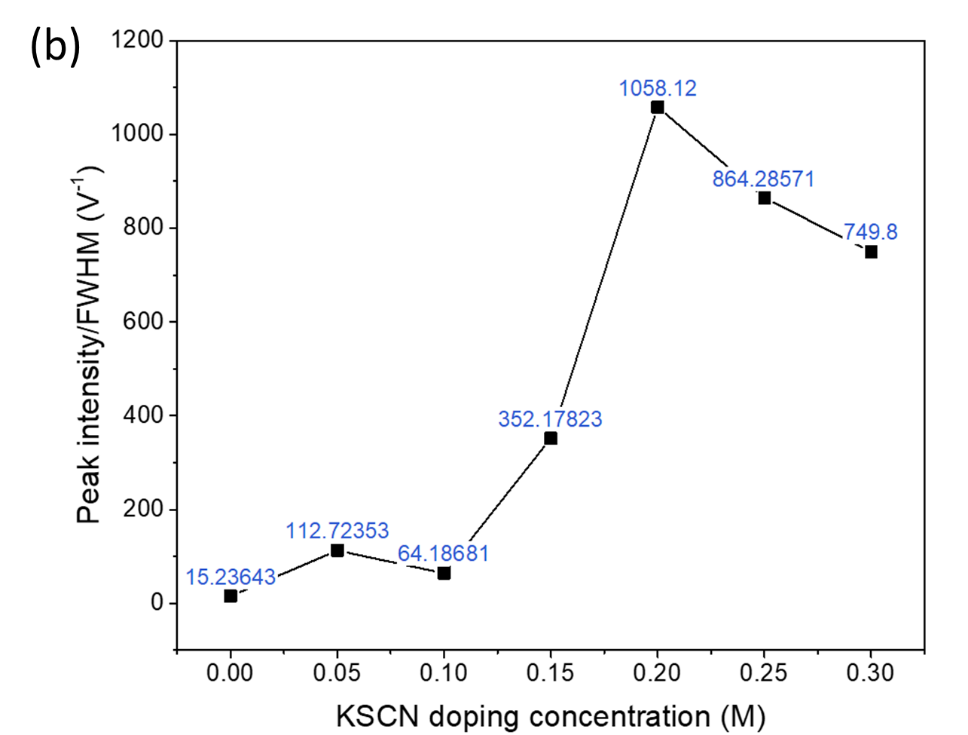


**Figure. S7.** **Surface potential characterization.** (a) Kelvin probe force microscopy (KPFM) and (b) surface potential distribution factor results from (a) of perovskite films deposited by MAPbI_3_ precursor solutions doped by KSCN in the concentration of from 0 to 0.30M with a 0.05M step on ITO.
